# Unilateral Crystalline Vitreoretinopathy: A Rare Entity Associated with Intraocular Inflammation

**DOI:** 10.1155/2015/460564

**Published:** 2015-11-24

**Authors:** Kaustubh B. Harshey, Karthik Srinivasan, Ramakrishnan Rengappa, Kim Ramasamy

**Affiliations:** ^1^Vitreo-Retina and Ocular Oncology, Sankara Eye Hospital, Kundalahalli Gate, Bangalore, Karnataka 560037, India; ^2^Aravind Eye Hospital and Postgraduate Institute of Ophthalmology, Tirunelveli, Tamil Nadu 627001, India; ^3^Aravind Eye Hospital and Postgraduate Institute of Ophthalmology, Madurai, Tamil Nadu 625020, India

## Abstract

A 31-year-old Indian male presented with floaters and diminution of vision in the right eye. Ocular examination showed features of old anterior uveitis with posterior subcapsular cataract and fine, refractile crystals in the vitreous cavity and on the retinal surface. A thorough workup for all known causes of crystalline retinopathy was inconclusive. Unilateral crystalline retinopathy has been sparingly reported. This is the first report of unilateral, crystalline vitreoretinopathy in the absence of any demonstrable and known cause for intraocular crystals.

## 1. Introduction

Crystalline retinopathies are known to be bilateral and are usually associated with an underlying metabolic disorder or drug intake [1]. Little is known and reported about unilateral crystalline retinopathies. We share the perplexing case of a young male who demonstrated features of unilateral crystalline vitreoretinopathy in the background of a healed anterior uveitis.

## 2. Case Presentation

**Figure 1:**
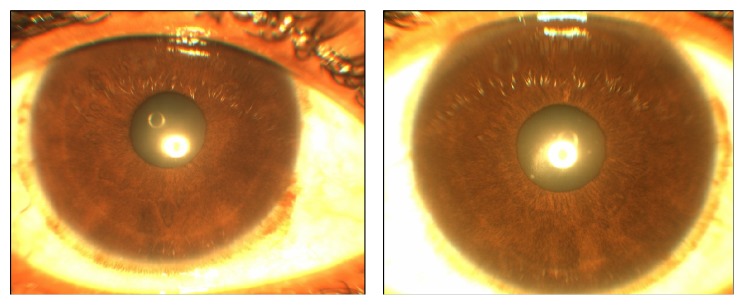
Anterior segment photograph showing subtle iris heterochromia (hypochromic right eye on the left side).

**Figure 2:**
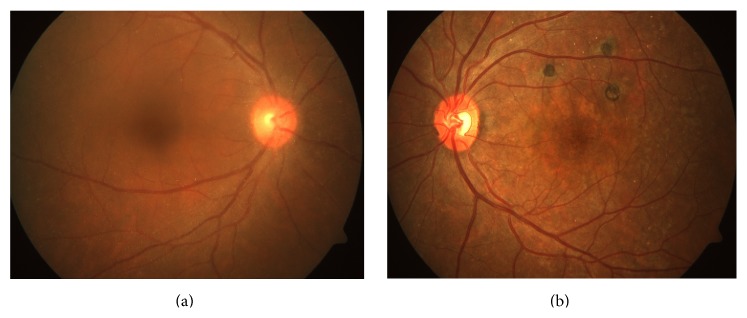
Fundus photograph prior to cataract surgery (right eye: (a)) showing crystal deposition over the retina. (b) Left eye for comparison.

**Figure 3:**
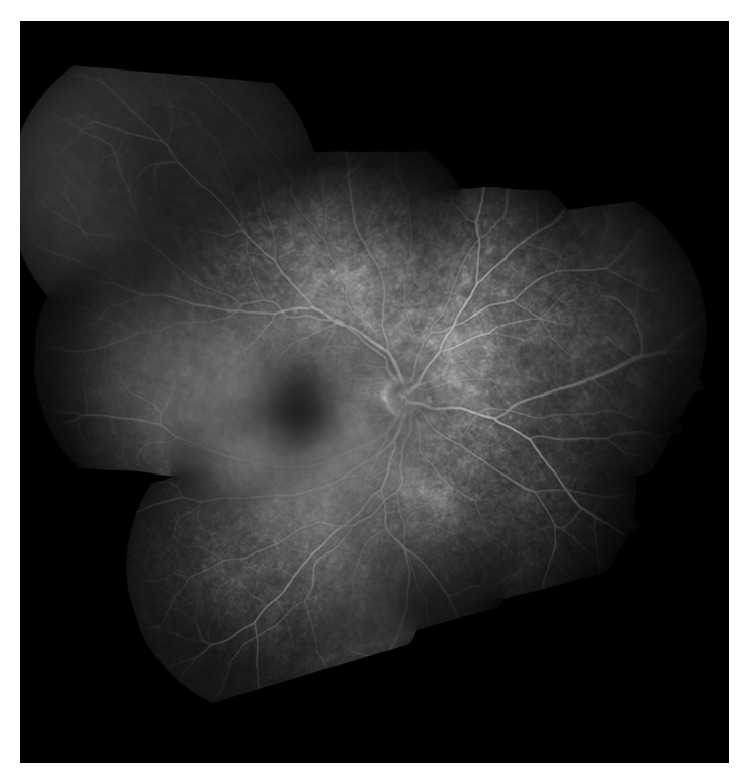
Fundus fluorescein angiogram of right eye (montage picture) showing normal retinal and choroidal fluorescence.

**Figure 4:**
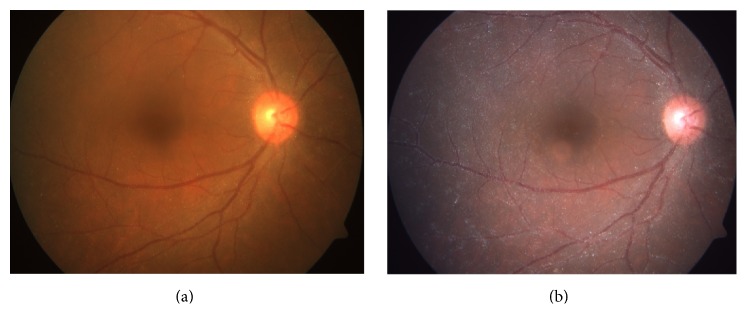
Fundus photograph post cataract surgery showing an apparent increase in crystal deposition ((a) preoperative picture for comparison).

**Figure 5:**
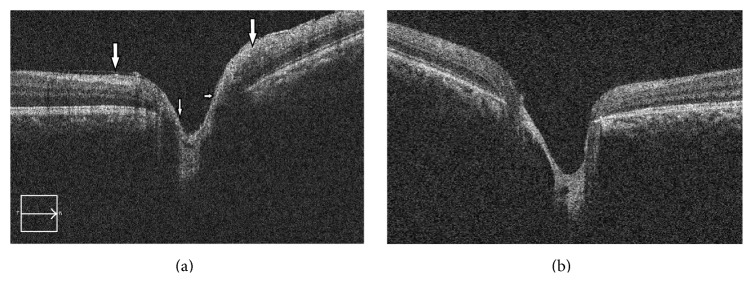
Optical coherence tomography through the optic nerve head showing hyperreflective crystals on the ILM and the inner retinal layers in the right eye ((b) left eye for comparison).

**Figure 6:**
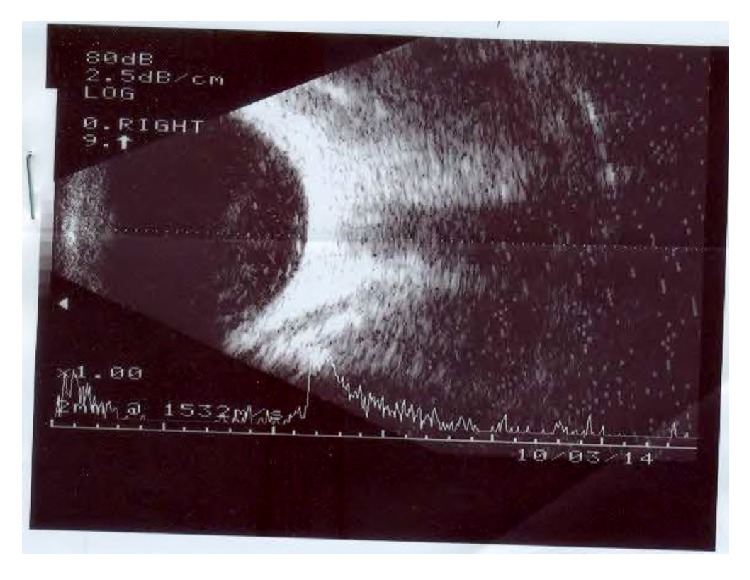
Ultrasound B-scan of the right eye showing moderately reflective echoes in the posterior vitreous without clear space separating it from the retinal surface.

A 31-year-old male presented with painless decrease in vision of right eye (RE) with floaters since one year. Best corrected visual acuity (BCVA) was 6/9 in RE and 6/6 in left eye (LE). The RE showed few pigmented, stellate keratic precipitates (KPs). Iris heterochromia was noted (Figure 1) along with posterior subcapsular cataract. Posterior segment revealed vitritis 1+ and fine, floating refractile crystals in the vitreous. Similar crystals were scattered over the retina, predominantly on the posterior pole sparing the fovea. They were superficial and preferentially distributed over the major retinal vessels and the optic disc (Figure 2). The patient denied receiving intravitreal injections, any history suggestive of recurrent ocular inflammation, or a history of trauma. Posterior segment of the LE showed a normal vitreous with patches of pigment proliferation and pigment epithelial mottling at the macula with few hard drusen. Fundus fluorescein angiogram (FFA) of RE was normal (Figure 3). The patient underwent phacoemulsification with foldable intraocular lens implantation. The BCVA in RE three months postoperatively was 6/6. The unusual crystal deposition appeared to have increased (Figure 4). Optical coherence tomography (OCT) of the RE showed hyperreflective lesions on the internal limiting membrane (ILM) and inner retinal layers (Figure 5). Ultrasound B-scan of RE showed moderately reflective mobile echoes in the vitreous without any clear space at the vitreoretinal interface (Figure 6). A total and differential leukocyte count, erythrocytic sedimentation rate, blood glucose, urinalysis, lipid profile, blood urea, and serum creatinine were within normal limits. Additionally, the patient was investigated for unilateral uveitis. A Mantoux test, chest X-ray, titers for syphilis, antinuclear antibodies, and serum acetylcholinesterase (ACE) were negative. As the patient was asymptomatic, he is currently under close observation.

## 3. Discussion

Crystalline retinopathy occurs commonly due to systemic drug toxicity or metabolic disorders [1]. In both, it is bilateral and often associated with loss of visual function. Our case presented uniquely with unilateral affection without visual loss which is previously unreported. Sarraf et al. [2] and Zarifa et al. [3] documented unilateral crystalline maculopathy secondary to intravitreal triamcinolone with superficial, refractile, white or yellow-green crystals, asymmetric in distribution, deposited in the central macula or around the fovea. OCT localized these to the posterior hyaloid surface. In contrast, our patient, without history of intravitreal injections, demonstrated diffuse crystals in the vitreous and the retina sparing the fovea with tendency to follow the vessels (Figure 2). OCT localized the deposits to the ILM and inner retina (Figure 5).

Three key features distinguish our case: no significant history, unilateral affection, and lack of visual loss due to crystals.

Hereditary causes were ruled out as family history for unexplained visual loss or heritable ocular disease was negative. Both Bietti's corneoretinal dystrophy and Sjogren Larsson syndrome are progressive conditions with significant visual dysfunction and other associated features. Cystinosis and hyperoxaluria present with features of renal disease. Secondary hyperoxaluria may present in adulthood but the crystals are deposited deeper in the retina [1]. Drug induced retinopathy was similarly ruled out in the absence of any history of intake of such drugs.

Crystal deposition has been demonstrated over the iris as Russell bodies in Fuchs' uveitis but not in the vitreous or retina [4]. Our case could be a case of Fuchs' uveitis but the KPs were few and heterochromia was subtle. There were no anterior segment crystals in our patient. A normal FFA ruled out breach of the blood-retinal barrier and also any vascular cause. The B-scan without any clear intervening space at the vitreoretinal surface also excluded asteroid hyalosis (Figure 6). Hence, in the absence of suggestive pathogenetic mechanism or diagnostic investigation the cause for crystal deposition remains obscure.

The purpose of this report was to highlight this singular, previously unreported occurrence of unilateral, crystalline vitreoretinopathy in the foreground of intraocular inflammation. This report will underline the presence of unilateral crystals without visual loss in the absence of known causes for crystalline retinopathy.
